# Comprehensive multi-method analysis of blood heavy metals and nutrient intake in myopia and high myopia

**DOI:** 10.1186/s12967-026-08086-1

**Published:** 2026-04-03

**Authors:** Jingwen Hui, Xinyuan Feng, Quanhong Han, Xuehao Cui

**Affiliations:** 1 Tianjin Eye Hospital, No.4 Gansu Road, Heping District, Tianjin, 300020 China; 2 Tianjin Key Lab of Ophthalmology and Visual Science, Tianjin, China; 3 School of Medicine, Nankai University, Tianjin, China; 4 Department of Clinical Neuroscience, University of Cambridge, Cambridge, UK

**Keywords:** Myopia, High myopia, Heavy metals, Vitamin B6, Nutritional epidemiology, Mendelian randomization, Machine learning, NHANES

## Abstract

**Background:**

High myopia constitutes a significant global public health concern. Although visual behaviors are well-established determinants, the relationships between systemic environmental exposures, nutritional factors, and high myopia remain incompletely characterized at the population level.

**Methods:**

We conducted a cross-sectional analysis of 3,283 adults from the National Health and Nutrition Examination Survey (NHANES) 1999–2008. Participants were categorized as healthy controls, non-high myopia, or high myopia based on non-cycloplegic autorefraction. Weighted multivariable logistic regression and restricted cubic spline models, as well as stratified and quantile analyses, were employed to assess exposure–outcome associations. Bayesian Kernel Machine Regression (BKMR) was applied to examine joint exposure patterns among correlated metals and dietary factors. Additionally, machine learning approaches (LASSO, Boruta, Elastic Net, and XGBoost) were utilized for exploratory variable selection and risk stratification.

**Results:**

Elevated blood concentrations of cadmium and mercury were associated with increased odds of high myopia, whereas several dietary and biochemical indicators demonstrated inverse associations under specific analytical frameworks. Carbohydrate intake was positively associated with high myopia. Nonlinear relationships and joint exposure patterns were identified across models. However, these findings exhibited substantial uncertainty and variability between methods. Machine learning analyses consistently identified cadmium exposure and carbohydrate intake as influential predictors, although variable importance rankings differed across algorithms and were interpreted as exploratory.

**Conclusions:**

In this nationally representative adult population, environmental metal exposures and nutritional factors were cross-sectionally associated with high myopia status. Given the cross-sectional design, the limited number of high myopia cases, potential measurement constraints, and residual confounding from unmeasured behavioral factors, these findings should be considered hypothesis-generating rather than indicative of causal or preventive relationships. Further longitudinal studies incorporating life-course exposure assessment and comprehensive behavioral data are warranted to clarify temporal dynamics and underlying biological mechanisms.

**Clinical trials registration:**

Not applicable.

**Supplementary Information:**

The online version contains supplementary material available at 10.1186/s12967-026-08086-1.

## Introduction

Myopia, particularly high myopia, has emerged as an escalating global public health concern [1]. By 2050, nearly half the global population will be affected by myopia, with approximately one billion individuals developing high myopia. This trend substantially increases the risks of sight-threatening complications, including retinal detachment, glaucoma, cataracts, and myopic maculopathy [2–4]. The rapid rise in myopia prevalence has largely been attributed to environmental changes, particularly increased near-work activities and reduced time spent outdoors [5]. However, behavioral factors alone do not fully explain interindividual variability in susceptibility, prompting growing interest in additional systemic determinants, including nutritional status and environmental exposures [6].

Emerging evidence suggests that dietary composition and nutritional status may play important roles in ocular growth and myopia development [7]. High-glycemic diets, characterized by rapid carbohydrate absorption, can elevate insulin and insulin-like growth factor-1 (IGF-1) levels, potentially promoting excessive axial elongation [8, 9]. Recent epidemiological studies have reported associations between high carbohydrate and sodium intake and increased myopia prevalence, whereas greater intake of omega-3 fatty acids, retinol, and certain vitamins appears to confer protective effects [10, 11]. Among these, B vitamins, particularly riboflavin (B2) and pyridoxine (B6), serve as essential cofactors in metabolic and neurotransmitter pathways critical for ocular tissue homeostasis. However, their roles in myopia remain insufficiently understood [12, 13]. In parallel, chronic exposure to environmental toxicants, especially heavy metals such as cadmium and lead, has been implicated in ocular pathology through mechanisms involving oxidative stress, retinal damage, and disruptions in scleral extracellular matrix homeostasis [14–16]. Cadmium, in particular, has been shown to induce inflammation and oxidative damage in retinal tissues. Yet its potential contribution to myopia pathogenesis has received limited attention [17].

Research on myopia risk has predominantly focused on visual behaviors and genetic predisposition, with relatively limited integration of nutritional and environmental determinants [5, 18]. Furthermore, studies examining single nutrients in isolation may fail to capture the complex interactions among dietary components and co-exposures to environmental factors [19]. Accordingly, a comprehensive analytical framework that simultaneously evaluates multiple nutritional biomarkers and trace metal exposures is warranted [20]. In order to address these gaps, we utilized data from the National Health and Nutrition Examination Survey (NHANES), a nationally representative dataset, to investigate associations between dietary nutrients, vitamins, trace metals, and myopia status (healthy control, non-high myopia, and high myopia) [21]. We employed an integrative analytical strategy incorporating multivariable regression, stratified subgroup analyses, restricted cubic spline modeling to assess nonlinear relationships, Bayesian kernel machine regression to evaluate mixture and interaction effects, and machine learning approaches for variable selection and predictive modeling. Additionally, Mendelian randomization (MR) was conducted to explore potential causal relationships. We hypothesized that specific dietary factors and metal exposures would exhibit systematic cross-sectional associations with myopia status, and that these associations may be consistent with, though not definitive evidence of, underlying biological mechanisms suggested by prior experimental and epidemiological studies. Through this multifaceted approach, we aimed to identify potentially modifiable systemic factors to inform targeted prevention strategies to mitigate the growing global burden of high myopia.

## Methods

### Study design and data source

We conducted a cross-sectional analysis using data from the NHANES, a nationally representative program employing a complex, multistage probability sampling method. Data were pooled from the 1999–2008 NHANES cycles, which included comprehensive ophthalmic and laboratory assessments. Participants aged ≥ 20 years with valid autorefractor-based refractive measurements (non-cycloplegic) were included. Individuals with hyperopia (spherical equivalent > + 0.50 diopters), a history of refractive or cataract surgery, or incomplete demographic or exposure data were excluded to minimize misclassification and bias. A total of 3,283 participants were included and categorized into three groups based on refractive status: (1) healthy controls (no myopia; spherical equivalent > − 0.50 diopters), (2) non-high myopia (mild-to-moderate myopia, ≤ − 0.50 diopters and > − 6.00 diopters), and (3) high myopia (≤ − 6.00 diopters in at least one eye). High myopia was defined as ≤ − 6.00 diopters, consistent with established clinical research criteria associated with an increased ocular morbidity. Because refractive error in NHANES was assessed using non-cycloplegic autorefraction, which may introduce accommodation-related misclassification, particularly in younger adults, we conducted age-restricted sensitivity analyses. Fully adjusted models were re-estimated after restricting the sample to participants aged ≥ 40 years and ≥ 50 years, respectively. Age was retained as a continuous covariate within these restricted samples to account for residual age-related variation. Consistency in the direction and magnitude of associations across these age thresholds was interpreted as supporting the robustness of findings, rather than evidence of causality.

### Ethical considerations

All NHANES protocols were approved by the National Center for Health Statistics Institutional Review Board, and written informed consent was obtained from participants. This present study was a secondary analysis of de-identified, publicly available data and was therefore exempt from additional institutional ethical review.

### Baseline data and exposure measurements

Demographic characteristics, including age, sex, race/ethnicity, and educational attainment, were obtained using standardized NHANES questionnaires. Lifestyle factors, including smoking and alcohol consumption, as well as the medical history of hypertension and diabetes, were also collected. Body Mass Index (BMI) was calculated from objectively measured height and weight. Visual acuity was assessed and converted to logarithm of the minimum angle of resolution (logMAR) units. Primary exposures of interest included blood concentrations of heavy metals (lead, cadmium, and mercury), measured by atomic absorption spectroscopy. Serum electrolytes and minerals (iron, potassium, sodium, phosphorus, and calcium) were measured through standardized biochemical assays. Dietary intake data, including macronutrients (total energy, protein, carbohydrates, fat, dietary fiber, and caffeine) and vitamins (A, B1, B2, B6, B12, and C), were obtained from 24-hour dietary recall interviews and adjusted for total energy intake. Blood-based nutritional biomarkers were not consistently available across all NHANES cycles or were not statistically significant in preliminary analyses. Therefore, our study focused on the exposures described above.

### Statistical analyses

All statistical analyses incorporated NHANES sampling weights and accounted for the complex survey design using Taylor series linearization. Statistical significance was initially defined as a two-tailed α < 0.05. Given the large number of exposures evaluated, we additionally controlled the false discovery rate (FDR) for the primary associations between metals, dietary factors, and high myopia using the Benjamini–Hochberg procedure. Corresponding FDR-adjusted p-values are reported in the tables. Baseline characteristics across healthy controls, non-high myopia, and high myopia groups were compared using weighted linear regression or analysis of variance (ANOVA) for continuous variables (mean ± SD) and Rao–Scott χ² tests for categorical variables. Missing data were addressed using a complete-case analysis approach. Participants with missing values for outcome status, exposure variables, or key covariates were excluded from the respective analyses. This approach was adopted due to the relatively low proportion of missing data and the limited number of high myopia cases, which could render multiple imputation unstable or highly model-dependent. Accordingly, all regression, spline, mixture, and machine learning analyses were conducted on complete observations for the variables included in each model.

### Multicollinearity assessment

Prior to fitting multivariable regression models, variance inflation factors (VIFs) were calculated for all candidate exposure variables included in the fully adjusted models to evaluate potential multicollinearity. VIF analyses were performed separately for the comparisons of myopia versus healthy controls and high myopia versus healthy controls. In accordance with established epidemiological conventions, a VIF < 5 was considered indicative of low multicollinearity, while values between 5 and 10 were interpreted as moderate but acceptable. No variables exceeded these predefined thresholds. Therefore, all selected exposures were retained for subsequent analyses.

### Multivariable regression and age-restricted sensitivity analyses

Multivariable logistic regression models were employed to assess independent associations between exposures and high myopia. Three sequential models were specified: Model 1 (unadjusted), Model 2 (adjusted for age, sex, and race/ethnicity), and Model 3 (fully adjusted for age, sex, race/ethnicity, education, BMI, smoking status, alcohol consumption, hypertension, diabetes, and all selected exposures entered simultaneously). Results were reported as β coefficients with 95% confidence intervals (CIs) and P-values. Regression coefficients, ORs, and P values were presented to three decimal places, with P values < 0.001 reported as “<0.001.”

### Stratified, quantile, and nonlinear analyses

Stratified analyses were conducted to evaluate subgroup-specific associations according to age (< 50 vs. ≥ 50 years), sex, hypertension, and diabetes status. Statistical interactions were assessed by incorporating multiplicative interaction terms into the regression models. Quantile regression was applied to examine exposure effects across different points of the refractive error distribution, specifically at the 10th and 50th percentiles of spherical equivalent, with adjustment for relevant covariates. Restricted cubic spline (RCS) analyses were implemented within logistic regression models to flexibly characterize potential nonlinear relationships between continuous exposures and the risk of high myopia.

### Bayesian kernel machine regression (BKMR)

In this study, BKMR was employed to evaluate the joint and potentially interactive effects of correlated exposures, including blood cadmium, blood mercury, serum sodium and phosphorus, dietary fiber, vitamins B2 and B6, and carbohydrate intake. Given the relatively limited number of high myopia cases and the presence of substantial correlations among several exposures, BKMR was applied in an exploratory framework to characterize potential joint exposure patterns rather than to infer stable estimates of interaction effects. Accordingly, results were interpreted descriptively, with particular caution in the evaluation of interaction surfaces and nonlinear relationships. We specified a probit link for the binary outcome of high myopia and used a Gaussian kernel to flexibly model potentially non-linear and non-additive exposure–response relationships. Models were fitted using 50,000 Markov chain Monte Carlo (MCMC) iterations, with the first 10,000 iterations discarded as burn-in. Every 10th iteration was retained for posterior inference in order to reduce autocorrelation. Model convergence was assessed through visual inspection of trace plots and evaluation of effective sample sizes. BKMR outputs included univariate exposure-response functions, bivariate interaction surfaces, and posterior inclusion probabilities (PIPs), which were used to summarize the relative importance of individual exposures within the mixture. We performed sensitivity analyses by refitting models with alternative priors and by excluding individual exposures from the mixture to evaluate robustness. These analyses yielded qualitatively consistent results. Uncertainty in estimated exposure–response relationships was quantified using 95% Bayesian credible intervals derived from the posterior distribution. Univariate exposure-response curves are presented with pointwise 95% credible intervals to reflect estimation uncertainty, rather than for formal hypothesis testing.

### Machine learning–based feature selection and predictive modeling

To address potential instability in multivariable modeling and the presence of correlated predictors, a two-stage feature selection and modeling strategy was implemented. Candidate predictors were initially screened using Least Absolute Shrinkage and Selection Operator (LASSO) regression with 10-fold cross-validation, in conjunction with the Boruta algorithm. Only variables consistently retained by these methods were included in subsequent modeling.

The dataset was randomly partitioned into a training set (70%) and an internal test set (30%), stratified by high myopia status. Given the substantial class imbalance (144 high myopia cases, approximately 4.4% of the sample), inverse-frequency class weights were applied during the training of tree-based models to mitigate bias toward the majority class.

Elastic Net logistic regression and Extreme Gradient Boosting (XGBoost) models were developed using the selected features. Hyperparameters were optimized via grid search or random search combined with 5-fold cross-validation. Model performance was evaluated using out-of-fold (OOF) predictions. Discriminative ability was assessed by the area under the receiver operating characteristic curve (AUC) and precision-recall curve (PRAUC), overall predictive accuracy was evaluated using the Brier score, and model calibration was assessed using calibration slope and intercept. Feature importance in the XGBoost model was quantified using the gain metric. Additionally, SHapley Additive exPlanations (SHAP) values were calculated to improve interpretability. All machine learning analyses were conducted for exploratory risk stratification purposes and were not intended to support causal inference.

### Software

All statistical analyses were performed using R (version 4.2.2). The survey package (v4.1) was used for complex survey analysis, glmnet (v4.1) for LASSO, randomForest (v4.7) and xgboost (v1.5) for machine learning models, Boruta (v7.0) for feature selection, pROC (v1.18) for ROC analysis, and rmda (v1.6) for decision curve analysis. BKMR analyses were conducted using the BKMR package (v0.2.0). SHAP values for the XGBoost model were obtained using the shapley function in the fastshap package (v0.0.7). MR was performed using the TwoSampleMR (v0.5.6) and MRPRESSO (v1.0) packages in R. Additionally, Python (version 3.9) was used for data preprocessing and supplementary analyses, with scikit-learn (v1.0) employed for cross-validation procedures.

## Result

### Baseline characteristics of participants

Table 1 summarizes the baseline characteristics of the 3,283 participants included in this study, categorized into three groups: 1,330 healthy controls, 1,809 individuals with non-high myopia, and 144 individuals with high myopia. Participants with high myopia were significantly younger than those in the healthy control group (mean age: 45.3 years vs. 49.2 years; FDR < 0.001) and included a higher proportion of females (66.7% vs. 58.6%; FDR < 0.001). They also exhibited higher blood cadmium concentrations (0.78 µg/L vs. 0.48 µg/L; FDR < 0.001) and elevated mercury levels (1.88 µg/L vs. 1.50 µg/L; FDR = 0.056). In contrast, serum sodium levels (138.5 mmol/L vs. 139.1 mmol/L; FDR = 0.010) and phosphorus levels (1.18 mmol/L vs. 1.23 mmol/L; FDR = 0.008) were significantly lower among individuals with high myopia. Dietary patterns also differed between groups. Participants with high myopia reported higher carbohydrate intake (276 g/day vs. 252 g/day; FDR = 0.070), alongside lower intake of dietary fiber (13.3 g/day vs. 15.8 g/day; FDR = 0.008), vitamin B2 (1.84 mg/day vs. 2.16 mg/day; FDR = 0.042), and vitamin B6 (1.57 mg/day vs. 1.88 mg/day; FDR = 0.008). Overall, these findings suggest a dietary pattern characterized by relatively higher carbohydrate consumption and lower intake of selected micronutrients among individuals with high myopia.

**Table 1:** Baseline characteristics by myopia status

Phenotype	Overall	HC	Myopia		*P*_HMvsHC_	*P*_Overall_	FDR_HMvsHC_	FDR_Overall_
			M	HM				
**N**	3283	1330	1809	144				
**Age**	47.0 (14.3)	49.2 (14.1)	45.5 (14.3)	45.3 (12.8)	**0.002**	**< 0.001**	**0.010**	**< 0.001**
Gender					0.074	**< 0.001**	0.158	**< 0.001**
Male	1175 (35.8%)	551 (41.4%)	576 (31.8%)	48 (33.3%)				
Female	2108 (64.2%)	779 (58.6%)	1233 (68.2%)	96 (66.7%)				
**Race**					0.073	**0.031**	0.158	0.103
Non-Hispanic White	1859 (56.6%)	750 (56.4%)	1037 (57.3%)	72 (50.0%)				
Non-Hispanic Black	599 (18.2%)	263 (19.8%)	310 (17.1%)	26 (18.1%)				
Other Hispanic	137 (4.17%)	58 (4.36%)	66 (3.65%)	13 (9.03%)				
Mexican American	527 (16.1%)	195 (14.7%)	305 (16.9%)	27 (18.8%)				
Other	161 (4.90%)	64 (4.81%)	91 (5.03%)	6 (4.17%)				
Education					0.125	0.279	0.208	0.419
Less than 9th Grade	216 (6.58%)	91 (6.84%)	117 (6.47%)	8 (5.56%)				
9-11th Grade	344 (10.5%)	151 (11.4%)	179 (9.89%)	14 (9.72%)				
High School	772 (23.5%)	324 (24.4%)	422 (23.3%)	26 (18.1%)				
College	1053 (32.1%)	412 (31.0%)	597 (33.0%)	44 (30.6%)				
Over College	898 (27.4%)	352 (26.5%)	494 (27.3%)	52 (36.1%)				
Smoke					0.250	0.475	0.375	0.594
Never	1744 (53.1%)	704 (52.9%)	957 (52.9%)	83 (57.6%)				
Former	863 (26.3%)	363 (27.3%)	470 (26.0%)	30 (20.8%)				
Current	676 (20.6%)	263 (19.8%)	382 (21.1%)	31 (21.5%)				
Alcohol					**0.018**	0.088	0.068	0.172
Never	460 (14.0%)	172 (12.9%)	257 (14.2%)	31 (21.5%)				
Former	671 (20.4%)	275 (20.7%)	369 (20.4%)	27 (18.8%)				
Current	2152 (65.5%)	883 (66.4%)	1183 (65.4%)	86 (59.7%)				
**Hypertension**					0.059	**< 0.001**	0.158	**< 0.001**
No	1970 (60.0%)	752 (56.5%)	1149 (63.5%)	69 (47.9%)				
Yes	1313 (40.0%)	578 (43.5%)	660 (36.5%)	75 (52.1%)				
**DM**					0.944	**0.011**	0.982	0.055
No	2699 (82.2%)	1063 (79.9%)	1520 (84.0%)	116 (80.6%)				
Yes	584 (17.8%)	267 (20.1%)	289 (16.0%)	28 (19.4%)				
BMI	29.2 (6.55)	29.3 (6.24)	29.1 (6.75)	29.5 (6.90)	0.825	0.707	0.926	0.816
**LogMar**	0.06 (0.14)	0.06 (0.11)	0.06 (0.13)	0.15 (0.31)	**< 0.001**	**< 0.001**	**< 0.001**	**< 0.001**
Blood_Pb	1.68 (1.26)	1.68 (1.14)	1.66 (1.32)	1.86 (1.51)	0.079	0.180	0.158	0.284
**Blood_Cd**	0.52 (0.54)	0.48 (0.52)	0.53 (0.53)	0.78 (0.78)	**< 0.001**	**< 0.001**	**< 0.001**	**< 0.001**
**Blood_Hg**	1.56 (1.65)	1.50 (1.62)	1.58 (1.62)	1.88 (2.15)	**0.013**	**0.029**	0.056	0.103
Blood_Fe	83.8 (34.6)	84.2 (34.1)	83.5 (35.0)	84.8 (35.0)	0.832	0.830	0.926	0.889
Blood_K	3.97 (0.32)	3.96 (0.33)	3.98 (0.32)	4.01 (0.28)	0.072	0.053	0.158	0.159
**Blood_Na**	139.3 (2.25)	139.1 (2.27)	139.8 (2.22)	138.5 (2.41)	**0.002**	**< 0.001**	**0.010**	**< 0.001**
**Blood_P**	1.22 (0.18)	1.23 (0.17)	1.22 (0.18)	1.18 (0.17)	**0.001**	**0.004**	**0.008**	**0.042**
Blood_Ca	2.36 (0.10)	2.36 (0.09)	2.36 (0.09)	2.36 (0.11)	0.292	0.473	0.417	0.594
Energy	2116.8 (913.5)	2088.8 (894.3)	2132.2 (919.1)	2166.8 (1006.3)	0.326	0.321	0.445	0.459
Protein	80.5 (39.7)	80.0 (38.9)	80.9 (40.2)	80.2 (40.3)	0.949	0.816	0.982	0.889
**Carbohydrate**	259.8 (121.6)	252.2 (116.7)	262.2 (123.2)	276.2 (134.4)	**0.021**	**0.015**	0.070	0.064
**Dietary_fiber**	15.6 (9.03)	15.8 (8.93)	15.6 (9.17)	13.3 (7.74)	**0.001**	**0.006**	**0.008**	**0.042**
Total_fat	81.6 (43.8)	81.5 (43.7)	81.0 (42.9)	89.1 (55.2)	0.055	0.103	0.158	0.172
Caffeine	180.5 (235.4)	180.2 (214.7)	181.7 (240.3)	180.8 (333.2)	0.983	0.997	0.983	0.997
Vit_A	609.6 (572.4)	604.4 (520.8)	613.8 (616.0)	595.0 (444.6)	0.833	0.883	0.926	0.913
Vit_B1	1.62 (0.97)	1.64 (1.05)	1.61 (0.91)	1.58 (0.88)	0.512	0.688	0.668	0.816
**Vit_B2**	2.15 (1.15)	2.16 (1.08)	2.16 (1.21)	1.84 (0.98)	**0.001**	**0.005**	**0.008**	**0.042**
**Vit_B6**	1.84 (1.09)	1.88 (1.09)	1.83 (1.10)	1.57 (0.79)	**0.001**	**0.003**	**0.008**	**0.042**
Vit_B12	5.15 (8.12)	5.07 (4.94)	5.26 (9.86)	4.45 (7.44)	0.164	0.460	0.259	0.594
**Vit_C**	89.4 (95.9)	83.1 (86.3)	94.1 (103)	87.7 (90.4)	0.547	**0.007**	0.684	**0.042**

The distributions of key demographic, biochemical, and dietary variables are presented in Figure S1. Several exposure variables, including blood cadmium, blood mercury, dietary fiber, carbohydrate intake, and vitamins B2 and B6, exhibited right-skewed distributions, whereas age, body mass index, serum sodium, and phosphorus were more symmetrically distributed. In light of these distributional characteristics, subsequent analyses employed modeling approaches that do not rely on normality assumptions, including log-scale visualization, quantile-based analyses, and flexible nonlinear modeling, to reduce sensitivity to skewness and extreme values.

### Multivariable regression analysis

Prior to interpreting the multivariable regression results, VIFs were evaluated to assess potential multicollinearity among exposure variables included in the fully adjusted models. As presented in Supplementary Table S4, all VIF values were below the predefined threshold of concern (VIF < 5) in both the myopia and high myopia models, indicating that collinearity among metals, dietary factors, and biochemical markers was unlikely to materially bias coefficient estimates. Accordingly, all selected variables were retained in subsequent analyses. Multivariable logistic regression analyses (Table 2) evaluated independent associations between biomarkers, dietary factors, and high myopia following sequential adjustment. In the fully adjusted model, blood cadmium emerged as a prominent risk factor (β = 0.135; FDR < 0.001), indicating a positive cross-sectional association with high myopia status. Blood mercury is also independently associated with increased risk, although with a smaller effect size (β = 0.015; FDR = 0.044). In contrast, higher serum sodium (β = − 0.020; FDR < 0.001) and phosphorus levels (β = − 0.163; FDR = 0.012) were inversely associated with high myopia. Dietary analyses revealed that higher carbohydrate intake was positively associated with high myopia (FDR < 0.001). Conversely, dietary fiber, vitamin B2, and vitamin B6 intake showed inverse associations in fully adjusted models. However, these dietary estimates are based on single 24-hour recall data and should be interpreted cautiously due to potential measurement error and substantial within-person variability, particularly for micronutrient intake. It is important to note that these regression coefficients represent conditional associations within a specific modeling framework. They should not be interpreted as indicators of relative variable importance, nor as directly comparable with results from spline-based, quantile, or machine learning analyses.

**Table 2:** The association between biomarkers and myopia

Biomarker	Model 1		Model 2		Model 3	
	Beta (95% CI)	FDR	Beta	FDR	Beta	FDR
BLOOD_CD	0.088 (0.053, 0.124)	**< 0.001**	0.103 (0.068, 0.139)	**< 0.001**	0.135 (0.088, 0.181)	**< 0.001**
BLOOD_HG	0.014 (0.002, 0.025)	**0.044**	0.020 (0.008, 0.032)	**< 0.001**	0.015 (0.002, 0.027)	**0.044**
BLOOD_NA	−0.018 (−0.026,−0.009)	**< 0.001**	−0.011 (−0.020,−0.002)	**0.026**	−0.020 (−0.029,−0.011)	**< 0.001**
BLOOD_P	−0.162 (−0.272,−0.053)	**0.011**	−0.208 (−0.317,−0.098)	**< 0.001**	−0.163 (−0.274,−0.051)	**0.012**
CARBOHYDRATE	< 0.001 (< 0.001, < 0.001)	**0.011**	< 0.001 (< 0.001, < 0.001)	**0.014**	< 0.001 (< 0.001, < 0.001)	**< 0.001**
DIETARY_FIBER	−0.002 (−0.004,−0.000)	0.064	−0.002 (−0.004, 0.000)	0.109	−0.004 (−0.007,−0.002)	**0.007**
VIT_B2	−0.013 (−0.030, 0.004)	0.14	−0.007 (−0.024, 0.011)	0.47	−0.045 (−0.077,−0.013)	**0.014**
VIT_B6	−0.024 (−0.042,−0.007)	**0.014**	−0.019 (−0.038,−0.001)	0.061	−0.054 (−0.081,−0.028)	**< 0.001**

Model 1: Non-adjusted

Model 2: Adjust by age, gender, race

Model 3: Adjust for: age, sex, race, and all covariates

### Subgroup analyses

Subgroup analyses (Table 3) were conducted to evaluate potential effect modifications by age, sex, hypertension status, and diabetes status. Consistent with the primary findings, blood cadmium demonstrated a stable positive association with high myopia risk across all subgroups, with no evidence of statistically significant interaction effects. Blood mercury exhibited modestly stronger associations among participants aged < 50 years (β = 0.023; FDR = 0.069) and among females (β = 0.022; FDR = 0.034), suggesting possible differences in susceptibility. However, most interaction terms did not remain statistically significant after FDR correction. Dietary factors showed some subgroup-specific patterns. The positive association between carbohydrate intake and high myopia was more pronounced among females after FDR adjustment, indicating potential sex-specific dietary influences. Vitamin B6 intake continued to demonstrate inverse associations in certain subgroups. However, given the limitations of dietary assessment and reduced sample sizes in subgroup analyses, these findings should be interpreted cautiously and not as evidence of definitive subgroup-specific protective effects.

**Table 3:** Subgroup analyses of biomarkers associated with myopia

Biomarkers	Sub-Group	Model 1		*P*_*interaction*_	Model 2		*P*_*interaction*_
		Beta (95% CI)	*FDR*		Beta	FDR	
BLOOD_CD	Age < 50	0.095 (0.040, 0.150)	**< 0.001**	0.64	0.149 (0.086, 0.212	**< 0.001**	0.80
	Age ≥ 50	0.112 (0.066, 0.158)	**< 0.001**		0.158 (0.105, 0.211)	**< 0.001**	
	Male	0.114 (0.056, 0.173)	**< 0.001**	0.23	0.153 (0.087, 0.219)	**< 0.001**	0.19
	Female	0.069 (0.025, 0.113)	**0.029**		0.105 (0.052, 0.157)	**< 0.001**	
	Non-Hypertension	0.058 (0.010, 0.106)	0.083	0.051	0.104 (0.048, 0.160)	**< 0.001**	0.091
	Hypertension	0.130 (0.077, 0.183)	**< 0.001**		0.165 (0.106, 0.225	**< 0.001**	
	DM	0.090 (0.052, 0.128)	**< 0.001**	0.59	0.133 (0.086, 0.180)	**< 0.001**	0.57
	Non - DM	0.060 (−0.041, 0.161)	0.32		0.101 (−0.004, 0.206)	0.173	
BLOOD_HG	Age < 50	0.023 (0.005, 0.040)	0.069	0.33	0.025 (0.007, 0.043)	0.050	0.46
	Age ≥ 50	0.011 (−0.004, 0.027)	0.243		0.016 (0.000, 0.032)	0.16	
	Male	0.006 (−0.012, 0.024)	0.604	0.18	0.005 (−0.013, 0.024)	0.663	0.17
	Female	0.022 (0.007, 0.038)	**0.034**		0.022 (0.007, 0.038)	0.050	
	Non-Hypertension	0.016 (0.001, 0.031)	0.111	0.59	0.016 (0.001, 0.031)	0.14	0.63
	Hypertension	0.009 (−0.010, 0.029	0.439		0.010 (−0.009, 0.030)	0.384	
	Non - DM	0.010 (−0.003, 0.023)	0.196	0.22	0.010 (−0.003, 0.023)	0.187	0.18
	DM	0.031 (−0.000, 0.063)	0.628		0.034 (0.002, 0.065)	0.143	
BLOOD_NA	Age < 50	−0.017 (−0.030,−0.004)	0.0675	0.36	−0.019 (−0.032,−0.006)	0.050	0.25
	Age ≥ 50	−0.009 (−0.021, 0.003)	0.202		−0.009 (−0.020, 0.003)	0.208	
	Male	−0.006 (−0.021, 0.009)	0.566	0.12	−0.006 (−0.021, 0.009)	0.529	0.096
	Female	−0.020 (−0.031,−0.010)	**< 0.001**		−0.022 (−0.032,−0.011)	**< 0.001**	
	Non-Hypertension	−0.019 (−0.031,−0.008)	**0.029**	0.63	−0.020 (−0.032,−0.009)	**< 0.001**	0.71
	Hypertension	−0.015 (−0.028,−0.002)	0.086		−0.017 (−0.030,−0.004)	0.058	
	Non - DM	−0.016 (−0.026,−0.006)	**0.029**	0.096	−0.015 (−0.025,−0.006)	**0.035**	0.10
	DM	−0.033 (−0.052,−0.015)	**< 0.001**		−0.033 (−0.051,−0.014)	0.173	
BLOOD_P	Age < 50	−0.228 (−0.381,−0.076)	**0.034**	0.19	−0.219 (−0.372,−0.066)	0.050	0.25
	Age ≥ 50	−0.083 (−0.238, 0.072)	0.371		−0.091 (−0.246, 0.064)	0.340	
	Male	0.167 (−0.014, 0.348)	**< 0.001**	**< 0.001**	0.151 (−0.030, 0.331)	0.173	**< 0.001**
	Female	−0.410 (−0.548,−0.273)	**< 0.001**		−0.405 (−0.543,−0.267)	**< 0.001**	
	Non - Hypertension	−0.180 (−0.322,−0.038)	0.069	0.76	−0.173 (−0.314,−0.031)	0.077	0.86
	Hypertension	−0.145 (−0.318, 0.028)	**< 0.001**		−0.153 (−0.327, 0.020)	**< 0.001**	
	Non - DM	−0.192 (−0.314,−0.070)	**0.029**	0.21	−0.191 (−0.314,−0.069)	**0.035**	0.29
	DM	−0.015 (−0.263, 0.234)	0.92		−0.044 (−0.292, 0.204)	0.765	
CARBOHYDRATE	Age < 50	< 0.001 (< 0.001, < 0.001)	0.744	0.14	< 0.001 (< 0.001, < 0.001)	0.683	0.15
	Age ≥ 50	< 0.001 (< 0.001, < 0.001)	0.086		< 0.001 (< 0.001, < 0.001)	0.094	
	Male	< 0.001 (< 0.001, < 0.001)	0.64	**0.0021**	< 0.001 (< 0.001, < 0.001)	0.802	**0.0010**
	Female	< 0.001 (< 0.001, < 0.001)	**< 0.001**		< 0.001 (< 0.001, < 0.001)	**< 0.001**	
	Non-Hypertension	< 0.001 (< 0.001, < 0.001)	0.276	0.18	< 0.001 (< 0.001, < 0.001)	0.27	0.20
	Hypertension	< 0.001 (< 0.001, < 0.001)	**< 0.001**		< 0.001 (< 0.001, < 0.001)	0.058	
	Non - DM	< 0.001 (< 0.001, < 0.001)	0.202	**0.011**	< 0.001 (< 0.001, < 0.001)	0.176	**0.011**
	DM	< 0.001 (< 0.001, < 0.001)	**< 0.001**		< 0.001 (< 0.001, < 0.001)	**< 0.001**	
DIETARY_FIBER	Age < 50	-0.002 (-0.004, -0.000)	0.128	0.92	-0.002 (-0.005, 0.001)	0.176	0.97
	Age ≥ 50	-0.002 (-0.005, 0.001)	0.284		-0.002 (-0.005, 0.001)	0.198	
	Male	-0.002 (-0.005, 0.001)	0.306	0.65	-0.002 (-0.005, 0.001)	0.272	0.60
	Female	−0.001 (−0.004, 0.002)	0.579		−0.001 (−0.004, 0.002)	0.603	
	Non-Hypertension	−0.003 (−0.006,−0.000)	0.086	0.34	−0.003 (−0.006,−0.000)	0.365	0.35
	Hypertension	−0.001 (−0.005, 0.003)	0.171		−0.001 (−0.004, 0.003)	0.683	
	Non - DM	−0.004 (−0.006,−0.002)	**0.029**	**< 0.001**	−0.004 (−0.006,−0.002)	**0.035**	**< 0.001**
	DM	0.006 (0.001, 0.011)	0.087		0.006 (0.001, 0.012)	0.078	
VIT_B2	Age < 50	-0.032 (-0.055, -0.009)	0.058	**0.022**	-0.031 (-0.054, -0.008)	0.058	**0.027**
	Age ≥ 50	0.007 (-0.017, 0.031)	0.64		0.007 (-0.018, 0.031)	0.683	
	Male	0.001 (−0.023, 0.026)	0.92	0.73	−0.000 (−0.025, 0.024)	0.99	0.73
	Female	−0.005 (−0.030, 0.020)	0.744		−0.006 (−0.031, 0.019)	0.683	
	Non-Hypertension	−0.021 (−0.043, 0.001)	0.17	0.32	−0.019 (−0.041, 0.002)	**< 0.001**	0.39
	Hypertension	−0.004 (−0.031, 0.023)	0.815		−0.004 (−0.031, 0.023)	0.784	
	Non - DM	−0.020 (−0.038,−0.002)	0.11	0.060	−0.018 (−0.037,−0.000)	0.161	0.099
	DM	0.027 (−0.018, 0.073)	0.32		0.023 (−0.023, 0.069)	0.414	
VIT_B6	Age < 50	-0.045 (-0.069, -0.021)	**< 0.001**	**0.031**	-0.043 (-0.067, -0.019)	**< 0.001**	**0.033**
	Age ≥ 50	−0.006 (−0.032, 0.020)	0.715		−0.005 (−0.030, 0.021)	0.765	
	Male	−0.021 (−0.046, 0.005)	0.196	0.47	−0.022 (−0.048, 0.004)	**< 0.001**	0.41
	Female	−0.007 (−0.033, 0.019)	0.651		−0.007 (−0.033, 0.019)	0.683	
	Non-Hypertension	−0.029 (−0.052,−0.007)	0.069	0.59	−0.028 (−0.051,−0.006)	0.077	0.59
	Hypertension	−0.019 (−0.048, 0.009)	0.267		−0.018 (−0.047, 0.010)	0.292	
	Non - DM	−0.033 (−0.052,−0.014)	**< 0.001**	**0.022**	−0.032 (−0.051,−0.013)	**0.035**	**0.026**
	DM	0.028 (−0.021, 0.077)	0.339		0.028 (−0.021, 0.077)	0.36	

**Model 1**: Non-adjusted model

**Model 2**: Adjust: AGE; GENDER; RACE; EDUCATION; BMI; SMOKE; ALCOHOL; HYPERTENSION; DM; LOGMAR; BLOOD_PB; BLOOD_FE; BLOOD_K; BLOOD_CA; ENERGY; PROTEIN; TOTAL_FAT; CAFFEINE; VIT_A; VIT_B1; VIT_B12

### Quantile stratification of key exposures

**Fig. 1:**
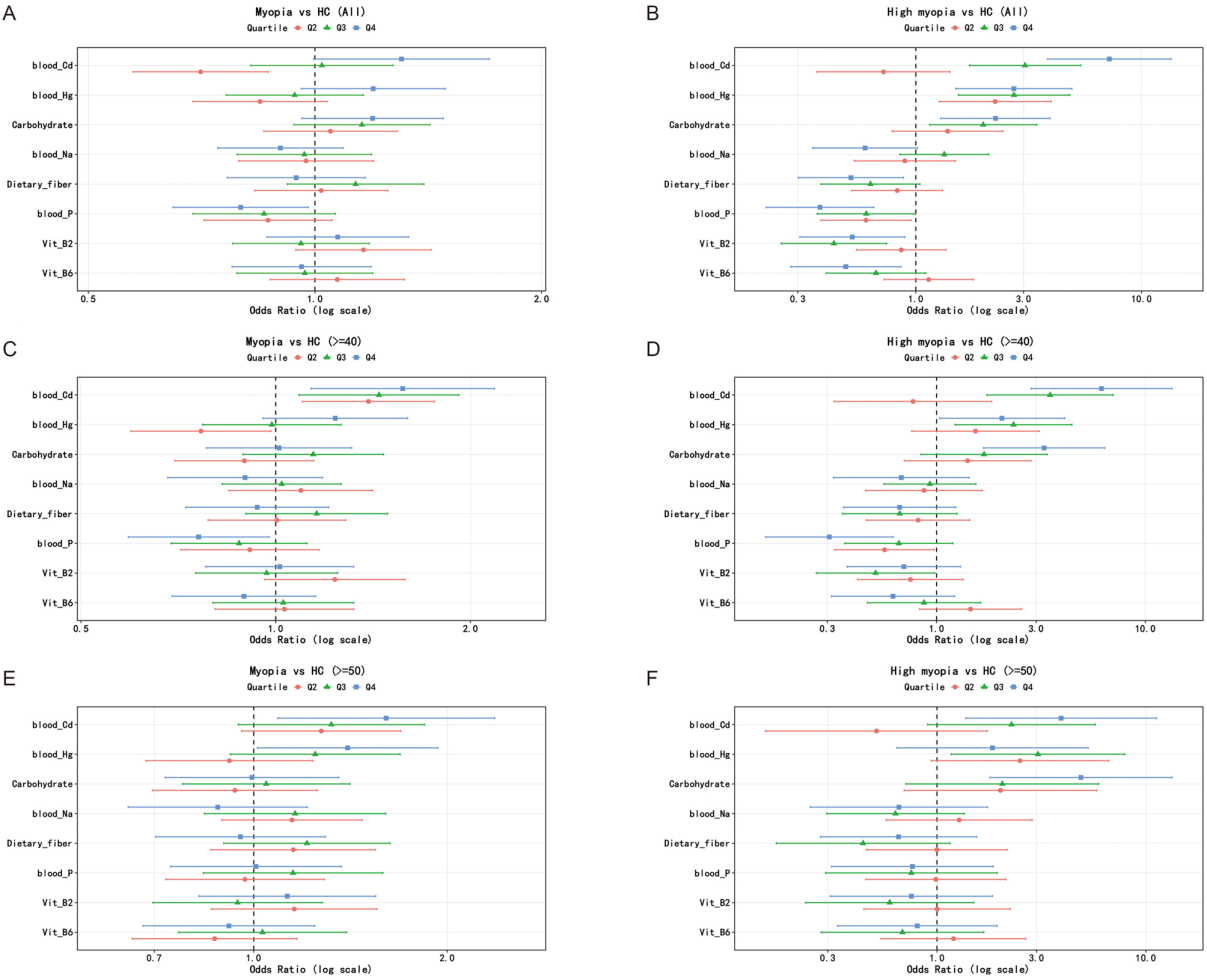
Associations of blood metals and dietary factors with myopia and high myopia. 1**A**–**F**. Forest plots showing adjusted odds ratios (ORs) and 95% confidence intervals (CIs) for quartiles of blood heavy metals and dietary factors in relation to myopia and high myopia. Quartile 1 (Q1) was used as the reference group. (**A**) Myopia versus healthy controls in the full sample. (**B**) High myopia versus healthy controls in the full sample. (**C**) Myopia versus healthy controls among participants aged ≥ 40 years. (**D**) High myopia versus healthy controls among participants aged ≥ 40 years. (**E**) Myopia versus healthy controls among participants aged ≥ 50 years. (**F**) High myopia versus healthy controls among participants aged ≥ 50 years. All models were adjusted for age, sex, race/ethnicity, education, body mass index, smoking status, alcohol consumption, hypertension, diabetes, and other relevant exposures. Odds ratios are presented on a logarithmic scale, with the dashed vertical line indicating OR = 1

Quantile stratification analyses demonstrated generally consistent dose-response patterns between key exposures and both myopia and high myopia across different age thresholds (Fig. 1; Table S1). In the overall adult population, higher quartiles of blood cadmium were associated with progressively increased odds of myopia and high myopia, with more pronounced differences observed in the upper exposure quartiles relative to the reference group. Notably, a similar directional pattern was observed in age-restricted analyses among participants aged ≥ 40 years and ≥ 50 years. However, given the limited number of high myopia cases, these quantile-based estimates should be interpreted as exploratory and may be sensitive to sampling variability.

For other exposures, including dietary fiber, carbohydrate intake, serum sodium, phosphorus, and vitamins B2 and B6, the direction of associations in quantile analyses was generally consistent with primary regression findings. Nevertheless, the magnitude and apparent importance of these associations varied across analytical approaches. Higher dietary fiber intake was associated with lower odds of high myopia in the quantile analyses. However, this observation should be interpreted cautiously due to reliance on single 24-hour dietary recalls, which may introduce measurement error and reflect correlated lifestyle factors rather than stable dietary effects. Although statistical significance varied across quartiles and age strata, the overall direction of associations remained broadly consistent. Taken together, these findings support a relatively robust cross-sectional association between blood cadmium levels and myopia status, while indicating more modest and potentially less stable associations for dietary and nutritional factors. These findings further support the robustness of the observed exposure-myopia relationships across different outcome definitions and age thresholds.

### Nonlinear dose–response relationships

**Fig. 2:**
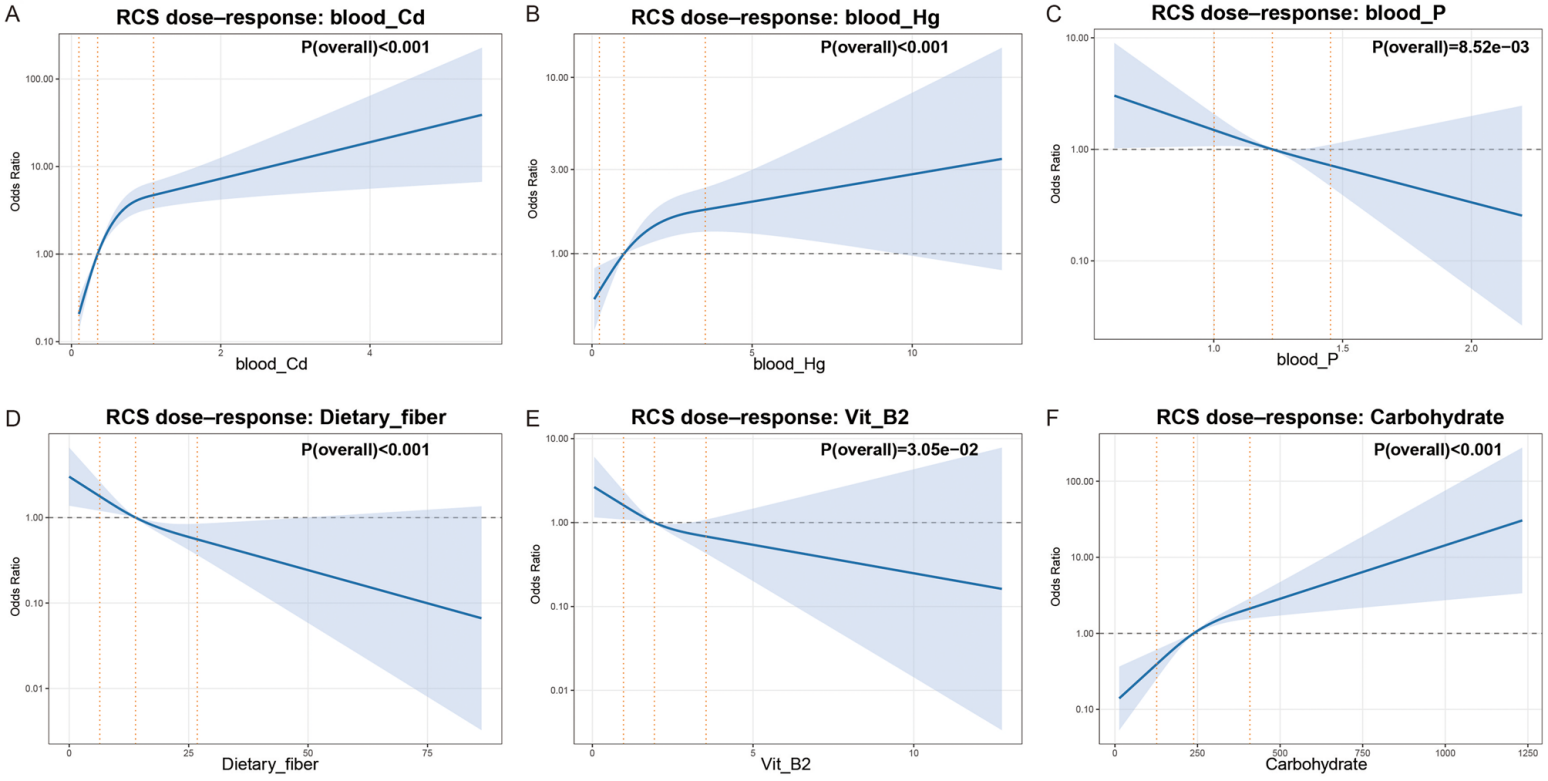
Restricted cubic spline analyses of dose-response relationships (primary analysis, K3). 2**A**–**F** Restricted cubic spline (RCS) analyses illustrating dose-response relationships between key blood metals, dietary factors, and the odds of high myopia, using three knots (10th, 50th, and 90th percentiles). (**A**) Blood cadmium (blood_Cd). (**B**) Blood mercury (blood_Hg). (**C**) Blood phosphorus (blood_P). (**D**) Dietary fiber intake. (**E**) Vitamin B2 intake (Vit_B2). (**F**) Carbohydrate intake. Solid blue lines represent adjusted odds ratios, and shaded areas indicate 95% confidence intervals. The horizontal dashed line denotes OR = 1. Vertical dotted lines indicate knot locations. The reference value corresponds to the median exposure level. P(overall) values from the Wald test are displayed in each panel

Restricted cubic spline analyses with three knots (K3) revealed nonlinear dose-response relationships between key exposures and the risk of high myopia (Fig. 2). Blood cadmium and mercury exhibited monotonic increases in risk, with more pronounced nonlinear changes observed at lower exposure levels. In contrast, serum phosphorus, dietary fiber, and vitamin B2 demonstrated inverse associations, with stronger effects apparent at lower exposure ranges that attenuated at higher levels. Carbohydrate intake showed a nonlinear positive association, characterized by a more marked increase in risk at higher intake levels. Sensitivity analyses using four knots (K4) produced qualitatively similar curve shapes and overall trends across all six exposures (Figure S2), supporting the robustness of these findings to knot specification.

### BKMR analysis of combined exposures

**Fig. 3:**
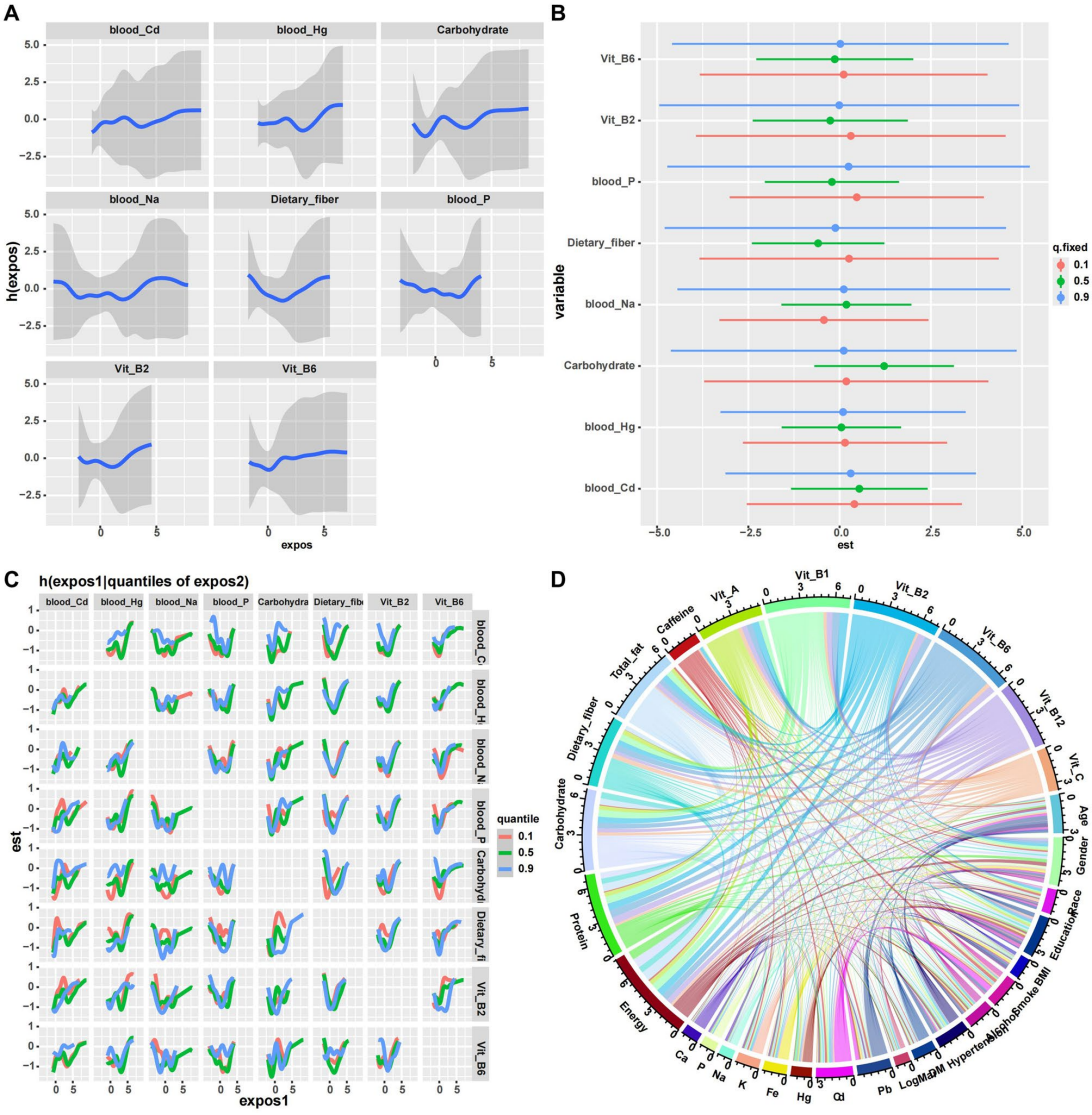
Mixture and interaction analyses using Bayesian Kernel Machine Regression (BKMR). 3**A**-**D** BKMR results evaluating joint, individual, and interactive effects of blood metals and dietary factors on high myopia. (**A**) Univariate exposure-response functions showing the estimated change in the outcome as each exposure varies while other exposures are held at their median levels. (**B**) Estimated overall effects of individual exposures at selected quantiles (0.1, 0.5, and 0.9) of the joint exposure distribution. (**C**) Bivariate exposure-response surfaces illustrating potential interaction patterns between selected exposure pairs. (**D**) Chord diagram depicting correlations and co-exposure structures among metals, nutrients, and covariates included in the BKMR model. Shaded areas represent 95% credible intervals. Results reflect posterior estimates from the fully adjusted BKMR model

BKMR analyses (Fig. 3A and D) were conducted to explore joint exposure patterns among correlated metals and dietary factors in relation to high myopia status (Table S2). Univariate exposure–response functions are presented with 95% credible intervals, which were relatively wide for several exposures, reflecting substantial uncertainty due to the limited number of outcome events and correlations among predictors. Bivariate exposure–response surfaces suggested potential non-additive joint patterns, particularly for combinations of blood cadmium with dietary fiber and blood mercury with vitamin B6. The carbohydrate–fiber surface illustrated differing risk profiles across intake combinations. However, given the limited number of high myopia cases and the degree of correlation among exposures, these apparent interaction patterns should be interpreted with caution, as they may be sensitive to modeling assumptions and could reflect statistical artifacts rather than reproducible biological interactions. The chord diagram (Fig. 3D) summarizes the correlation structure among exposures and is presented for descriptive purposes only, without implying causal or actionable relationships.

### Machine learning variable selection and nomogram construction

**Fig. 4:**
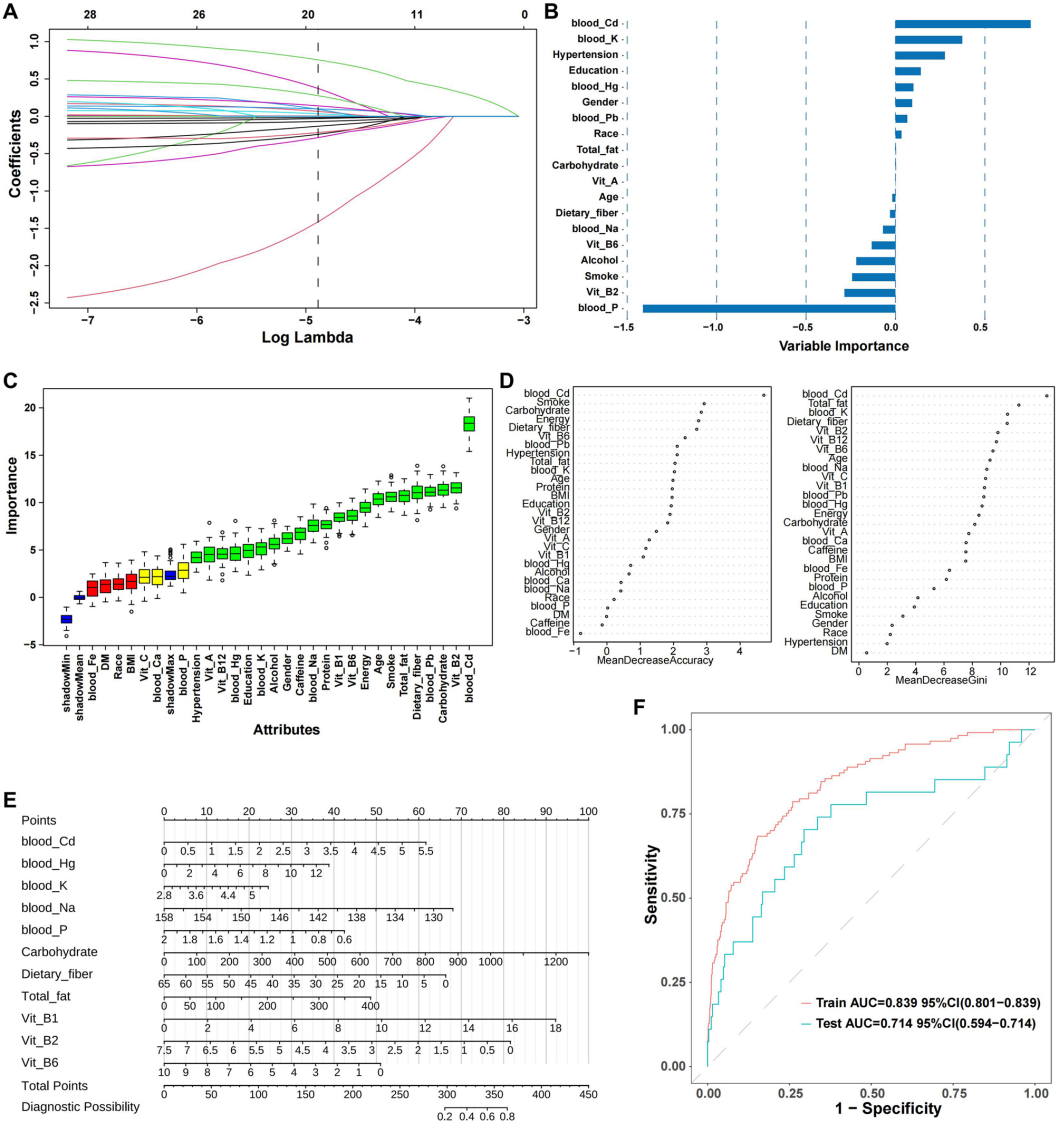
Feature selection and variable importance from machine learning models. 4**A**-**F** Machine learning-based feature selection and importance ranking for predictors of high myopia. (**A**) Elastic Net coefficient paths as a function of the regularization parameter (log λ). (**B**) Standardized coefficients of selected features from the Elastic Net model. (**C**) Boruta algorithm results showing confirmed, tentative, and rejected features based on importance scores. (**D**) Random Forest variable importance measured by mean decrease in accuracy and Gini index. (**E**) Nomogram constructed using selected predictors to estimate the probability of high myopia. (**F**) Receiver operating characteristic (ROC) curves for training and test datasets, illustrating model discrimination

Figure 4 summarizes the variable selection process across multiple machine learning approaches. Consistent findings from LASSO, Boruta, and Random Forest analyses (Fig. 4A–D) identified blood cadmium as the most influential predictor, alongside carbohydrate intake, dietary fiber, selected blood minerals, and vitamins. While certain variables, particularly blood cadmium, were consistently selected across methods, the relative importance and ranking of other predictors varied substantially between regression-based and machine learning approaches, reflecting methodological differences rather than true stability. Based on the selected variables, a nomogram was constructed to visualize their relative contributions to the predicted probability of high myopia (Fig. 4E). Higher blood cadmium concentrations were associated with increased predicted risk, whereas greater intake of dietary fiber and vitamin B6 was associated with lower predicted risk. This nomogram is intended for exploratory risk stratification and requires external validation prior to any potential clinical application.

### Machine learning model validation and interpretation

**Fig. 5:**
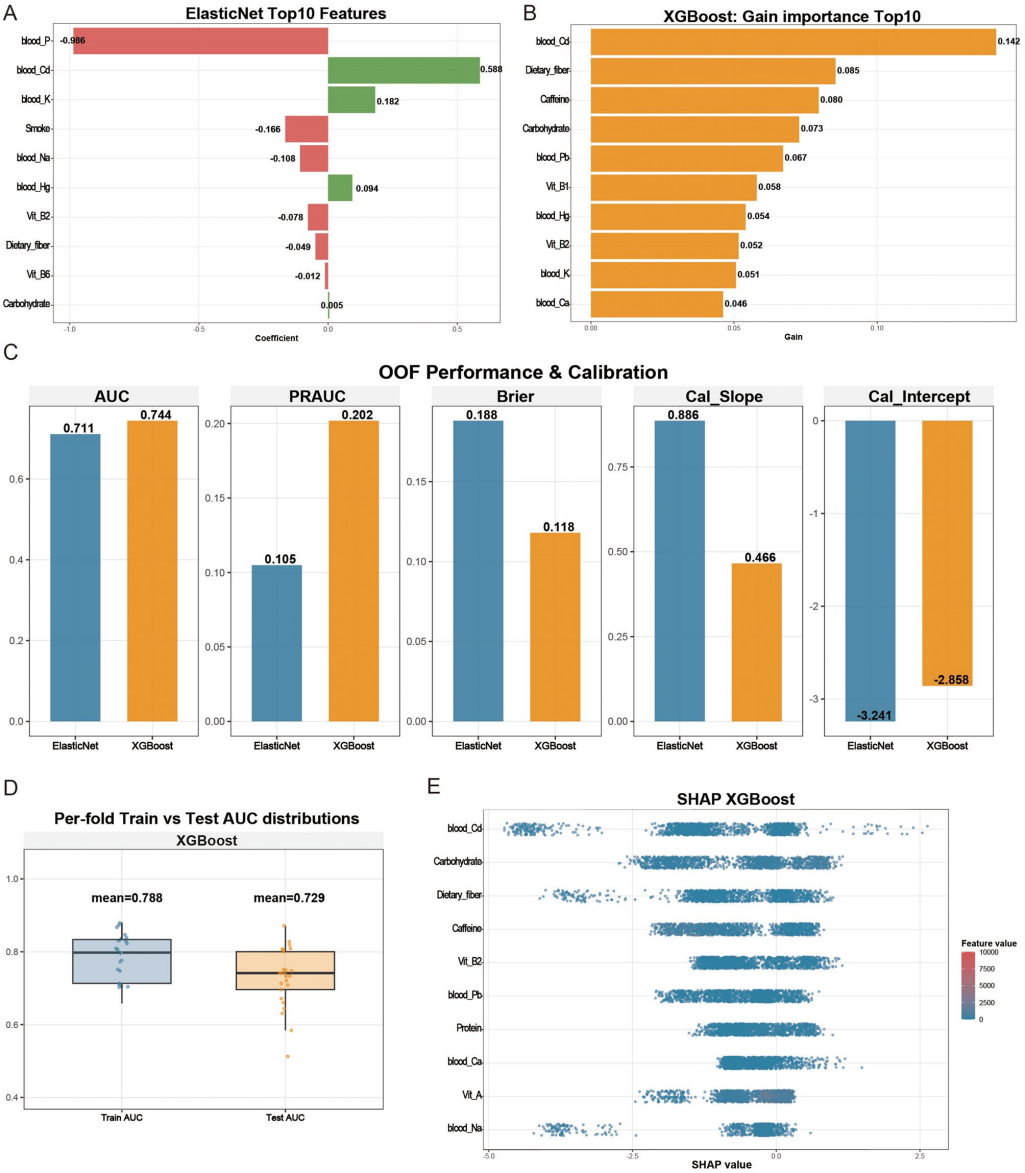
Predictive performance, calibration, and interpretation of machine learning models. 5**A**-**E** Comparison and interpretation of predictive models for high myopia. (**A**) Top 10 features identified by the Elastic Net model. (**B**) Top 10 features ranked by gaining importance from the XGBoost model. (**C**) Out-of-fold (OOF) performance metrics, including area under the ROC curve (AUC), precision-recall AUC (PRAUC), Brier score, calibration slope, and calibration intercept for Elastic Net and XGBoost models. (**D**) Distributions of training and test AUCs across cross-validation folds for the XGBoost model. (**E**) SHapley Additive exPlanations (SHAP) summary plot for the XGBoost model, illustrating the direction and magnitude of each feature’s contribution to predicted high myopia risk

Following feature preselection using LASSO regression and the Boruta algorithm, Elastic Net and XGBoost models were developed using a reduced and more stable set of predictors (Fig. 5A–B). Across both approaches, blood cadmium consistently emerged as the most influential predictor, along with dietary fiber, carbohydrate intake, and selected micronutrients and electrolytes. Model performance was evaluated using OOF predictions (Fig. 5C). XGBoost demonstrated modestly improved discrimination compared with Elastic Net (AUC 0.744 vs. 0.711) and superior performance under class imbalance, as indicated by a higher PRAUC (0.202 vs. 0.105) and a lower Brier score (0.118 vs. 0.188). Calibration was acceptable for both models, although XGBoost showed evidence of slight over-shrinkage. Per-fold comparisons of training and test AUCs for XGBoost (mean 0.788 vs. 0.729; Fig. 5D) suggested improved generalizability relative to earlier high-dimensional models, while indicating that some degree of residual overfitting cannot be entirely excluded. Feature importance rankings and SHAP analyses for XGBoost were directionally consistent (Fig. 5B and E), demonstrating positive contributions of higher blood cadmium and carbohydrate intake to predicted high myopia risk, and negative contributions of dietary fiber and vitamin-related features.

## Discussion

This study characterizes cross-sectional associations between selected environmental and nutritional exposures and high myopia status in adults. Blood cadmium and mercury were positively associated with high myopia, whereas several dietary and biochemical measures exhibited inverse associations. Although some analyses suggested nonlinear exposure–response patterns across the observed ranges, these findings should be interpreted with caution. Given the cross-sectional design and the limited number of high myopia cases, such patterns should not be used to infer thresholds, safety limits, causal mechanisms, or protective effects. In addition, while certain dietary variables demonstrated inverse associations in specific analytical frameworks, their relative importance and effect patterns were not consistent across methods. A key methodological consideration is the imbalance between the relatively small number of high myopia cases and the breadth of analytical approaches applied. This imbalance constrains statistical power and increases the potential for model instability, particularly for high-dimensional or flexible methods such as restricted cubic splines, quantile regression, Bayesian kernel machine regression, and machine learning models. Under these conditions, observed nonlinearities, interaction patterns, subgroup differences, and feature importance rankings may be sensitive to modeling assumptions and may not generalize beyond the present dataset. Although multiple analytical safeguards were implemented, residual instability cannot be excluded. Differences across methods are therefore expected, given their distinct assumptions and optimization objectives. Accordingly, no single analytical framework was considered definitive. Rather, findings were interpreted collectively as complementary and exploratory perspectives, rather than as a unified hierarchy or variable importance. An additional consideration is the joint modeling of dietary variables and metal exposures, which are inherently correlated. This correlation reflects real-world exposure structures rather than a methodological artifact, as diet represents both a primary source of metal exposure and a key determinant of nutritional status. Analyses that consider metals or dietary factors in isolation may therefore be subject to residual confounding by the complementary exposure domain. In this context, joint modeling was adopted to estimate conditional associations within a shared exposure framework, rather than to infer independent or separable causal pathways. This approach aligns with contemporary practices in environmental epidemiology, where co-exposures are regarded as an intrinsic feature of the exposure landscape rather than a nuisance to be eliminated.

The biological mechanisms underlying these observations warrant careful consideration. Heavy metals such as cadmium and mercury are well-recognized ocular toxicants [22, 23]. Cadmium can accumulate in retinal tissues, inducing oxidative stress and cellular injury, while mercury exerts comparable neurotoxic effects on the visual system [24]. Chronic oxidative stress and inflammation in the retina and sclera have been implicated in pathological axial elongation and are considered key processes in myopia progression [25, 26]. Nutrients with antioxidant or anti-inflammatory properties have been proposed, based on experimental and mechanistic evidence, to influence ocular physiology [27]. However, the present observational findings, based on single 24-hour dietary recalls, do not provide a sufficient basis for inferring such mechanisms or for establishing links between dietary intake and reduced myopia risk. Vitamin B6 serves as an essential cofactor in neurotransmitter synthesis and may play a role in regulating ocular growth [28, 29]. It has also been shown to quench reactive oxygen species, thereby mitigating oxidative stress [30]. Experimental studies have suggested that adequate vitamin B6 status may influence oxidative stress pathways in ocular tissues and modulate biochemical processes involved in axial length regulation [31]. Similarly, dietary fiber has been hypothesized to affect myopia risk through multiple pathways, including improved metabolic control and binding heavy metals in the gut, thereby limiting their systemic absorption [32]. The nonlinear dose–response relationships observed for dietary fiber and vitamin B6 are broadly consistent with these proposed mechanisms. Specifically, once intake reaches levels sufficient to meet metabolic demands or optimize antioxidant capacity, additional intake may confer diminishing marginal benefits, reflecting a potential saturation effect [33]. Nevertheless, the observed association patterns primarily indicate that differences in intake levels may co-occur with myopia status and do not permit inference regarding optimal intake thresholds or protective effects. In contrast, the inverse association observed between serum sodium and high myopia is less readily explained by current biological understanding. This finding may reflect residual confounding or the possibility that serum sodium serves as a proxy for other aspects of health status. Accordingly, this finding should be interpreted very carefully and requires replication in independent populations.

The interplay among multiple exposures was further examined using BKMR, which provides a flexible framework for visualizing joint patterns among correlated exposures. In the present study, BKMR surfaces suggested potential non-additive joint patterns between certain metals and dietary factors, such as cadmium with dietary fiber and mercury with vitamin B6. However, BKMR requires an adequate sample size and a well-characterized covariance structure to support stable inference, given the limited number of high myopia cases and the substantial correlations among exposures in this dataset. The observed interaction surfaces may be sensitive to kernel specification, prior assumptions, and sampling variability, and may therefore reflect modeling artifacts rather than reproducible biological interactions. Accordingly, these BKMR findings should be interpreted as exploratory visualizations that generate hypotheses for future investigation, rather than evidence of synergic, antagonistic, or mechanistic relationships.

These considerations extend to other high-dimensional and non-parametric analytical approaches employed in this study, including restricted cubic splines, quantile regression, Bayesian kernel machine regression, and machine learning classifiers. In the context of limited outcome events, such methods are inherently sensitive to modeling choices, such as tuning parameters, knot placement, and resampling strategies, and may yield unstable patterns that do not generalize beyond the analyzed dataset. Consequently, apparent nonlinear relationships, interaction structures, and feature importance rankings should be interpreted cautiously as exploratory signals rather than definitive associations. To partially mitigate these limitations, we implemented multiple methodological safeguards. These included variable preselection using LASSO regression and Boruta algorithm, assessment of multicollinearity, age-restricted sensitivity analyses, comparison of alternative spline specifications (K3 versus K4), and OOF evaluation with calibration metrics for machine learning models. Nevertheless, these measures cannot fully compensate for the limited number of high myopia cases, and residual instability in the estimates cannot be excluded.

We also observed heterogeneity across population subgroups, providing additional insight into potential effect modification. Mercury exposure demonstrated a stronger association with myopia risk among female participants, suggesting possible sex-related differences in exposure patterns or biological susceptibility [34, 35]. Additionally, metabolic health status appeared to modify the associations of nutritional factors with myopia. Among individuals without diabetes, higher intakes of dietary fiber and vitamin B6 were associated with lower myopia risk, whereas in those with diabetes, the inverse association with vitamin B6 appeared more pronounced [36, 37]. This pattern may reflect diabetes-related physiological changes, including chronic hyperglycemia and systemic inflammation, which could attenuate the metabolic benefits of dietary fiber while increasing the relative importance of nutrients such as vitamin B6 that support anti-inflammatory and metabolic pathways [38, 39]. Such subgroup-specific findings underscore the importance of considering individual factors, such as sex and metabolic health, when developing nutritional recommendations for the prevention of myopia.

From a clinical and public health perspective, these results primarily support hypothesis generation and exploratory risk stratification rather than immediate translation into practice. We utilized the identified risk and protective factors to develop a machine learning-based risk prediction model, presented as a nomogram to estimate the probability of high myopia at the individual level. The model demonstrated moderate discriminative performance in the internal test set (AUC > 0.7), indicating reasonable predictive ability within the study population. However, the model remains susceptible to overfitting and has not undergone external validation. Accordingly, the nomogram should be considered an exploratory tool for risk stratification rather than a clinically actionable instrument. Any targeted interventions aimed at reducing heavy metal exposure or optimizing nutritional intake should be considered experimental and grounded in general health promotion principles, rather than as established strategies for myopia prevention. This risk-guided, individualized framework may, if validated in independent cohorts and evaluated in interventional studies, eventually complement existing population-level approaches to myopia control.

### Study limitations

This study has several important limitations. First, the cross-sectional design of NHANES precludes causal inference. Myopia, particularly high myopia, typically develops during childhood and adolescence, whereas blood metal biomarkers and 24-hour dietary recalls reflect short-term or recent adult exposures. This temporal mismatch is especially problematic for micronutrients, for which single-day intake measures are poor proxies for habitual exposure and may yield unstable associations with chronic conditions such as myopia. Consequently, the biological interpretability of these findings is inherently limited and cannot be resolved through statistical adjustment. All results should therefore be interpreted strictly as cross-sectional associations rather than evidence of risk, causality, mechanisms, or prevention.

Second, several key determinants of myopia were not available in NHANES, including time spent outdoors, near-work intensity, screen exposure, and parental myopia. These factors are among the strongest predictors of myopia development, and their absence renders the observed associations highly susceptible to unmeasured confounding. This represents a fundamental limitation of the analysis.

Third, both exposure and outcome measurements are subject to error. Refractive error was assessed using a single non-cycloplegic autorefraction, which may introduce accommodation-related misclassification, particularly in adults under 50 years of age. Classification of high myopia is especially sensitive to such errors. Blood metal concentrations primarily reflect recent exposure rather than cumulative or early-life exposure, and dietary intake was derived from a single 24-hour recall, which is prone to recall bias and substantial day-to-day variability, especially for micronutrients.

Fourth, the number of high myopia cases was limited (approximately 144 events), constraining statistical power and model stability. The application of multiple high-dimensional and flexible methods, including multivariable regression, restricted cubic splines, quantile regression, Bayesian kernel machine regression, and machine learning, introduces the possibility that some observed patterns reflect modeling variability rather than underlying biological relationships. Although multiple safeguards were implemented, residual instability cannot be excluded.

Fifth, machine learning results should be interpreted with caution. Class imbalance and discrepancies between training and test performance suggest limited generalizability, and feature importance rankings should be regarded as exploratory rather than stable indicators of predictive relevance.

Sixth, particular caution is warranted for the interpretation of Bayesian kernel machine regression results. Given the limited number of outcome events and the presence of correlated exposures, apparent interaction patterns may reflect modeling artifacts rather than reproducible biological signals. Accordingly, BKMR findings are presented descriptively and should be viewed as hypothesis-generating.

Finally, the generalizability of these findings is limited. The analysis was restricted to U.S. adults from earlier NHANES cycles and may not extend to children or adolescents, among whom myopia typically develops, or to populations with different genetic, environmental, and lifestyle contexts. Overall, these limitations indicate that the present findings should be interpreted as exploratory and hypothesis-generating, warranting confirmation in larger, longitudinal studies with life-course exposure assessment and comprehensive measurement of behavioral determinants.

## Conclusion

In conclusion, our study identifies cross-sectional associations between environmental heavy metals, nutritional factors, and the risk of high myopia, generating hypotheses for future investigation. Higher blood concentrations of cadmium and mercury were consistently associated with increased odds of high myopia, whereas dietary fiber and vitamin B6 intake demonstrated inverse associations in selected analytical frameworks. These findings should be interpreted as hypothesis-generating observations that require confirmation in larger, longitudinal studies with sufficient outcome events and life-course exposure assessment. Ultimately, if validated through prospective and experimental research, these observations may contribute to a broader understanding of the role of systemic exposures in myopia. However, the current evidence alone does not support preventive or clinical recommendations, warranting further research.

## Supplementary Information

Below is the link to the electronic supplementary material.

Figure S1. Distributional characteristics of key variables. Histograms, kernel density plots, and quantile-quantile (Q-Q) plots illustrating the distributions of demographic variables, refractive measures, blood metals, and dietary factors across healthy controls, myopia, and high myopia groups. These plots were used to assess skewness, outliers, and deviations from normality prior to modeling

Figure S2. Sensitivity analyses of restricted cubic spline models (K4). Sensitivity analyses of dose-response relationships using restricted cubic splines with four knots (5th, 35th, 65th, and 95th percentiles). (A) Blood cadmium (blood_Cd). (B) Blood mercury (blood_Hg). (C) Blood phosphorus (blood_P). (D) Carbohydrate intake. (E) Dietary fiber intake. (F) Vitamin B2 intake (Vit_B2)

Supplementary Material 3

Supplementary Material 4

Supplementary Material 5

Supplementary Material 6

## Data Availability

All data supporting this study are included in this article and its supplementary materials.
