# Supplementary figures and images for: Comprehensive multi-method analysis of blood heavy metals and nutrient intake in myopia and high myopia
Source: J Transl Med. 2026 Apr 3;24:674. doi: 10.1186/s12967-026-08086-1 (PMC13170305; doi:10.1186/s12967-026-08086-1)

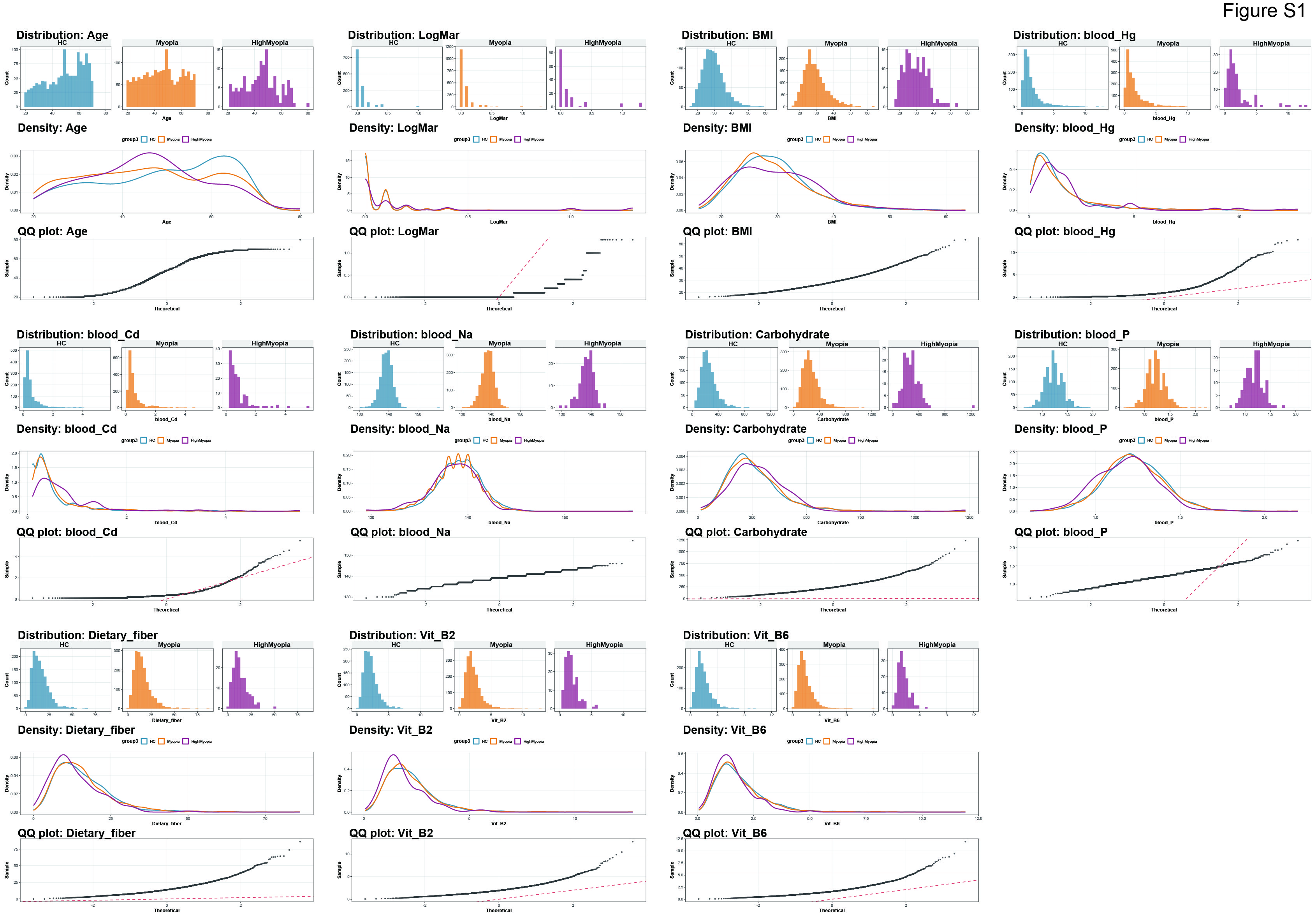

Supplement: Supplementary file 1 — Figure S1. Distributional characteristics of key variables. Histograms, kernel density plots, and quantile-quantile (Q-Q) plots illustrating the distributions of demographic variables, refractive measures, blood metals, and dietary factors across healthy controls, myopia, and high myopia groups. These plots were used to assess skewness, outliers, and deviations from normality prior to modeling [file 12967_2026_8086_MOESM1_ESM.jpg]

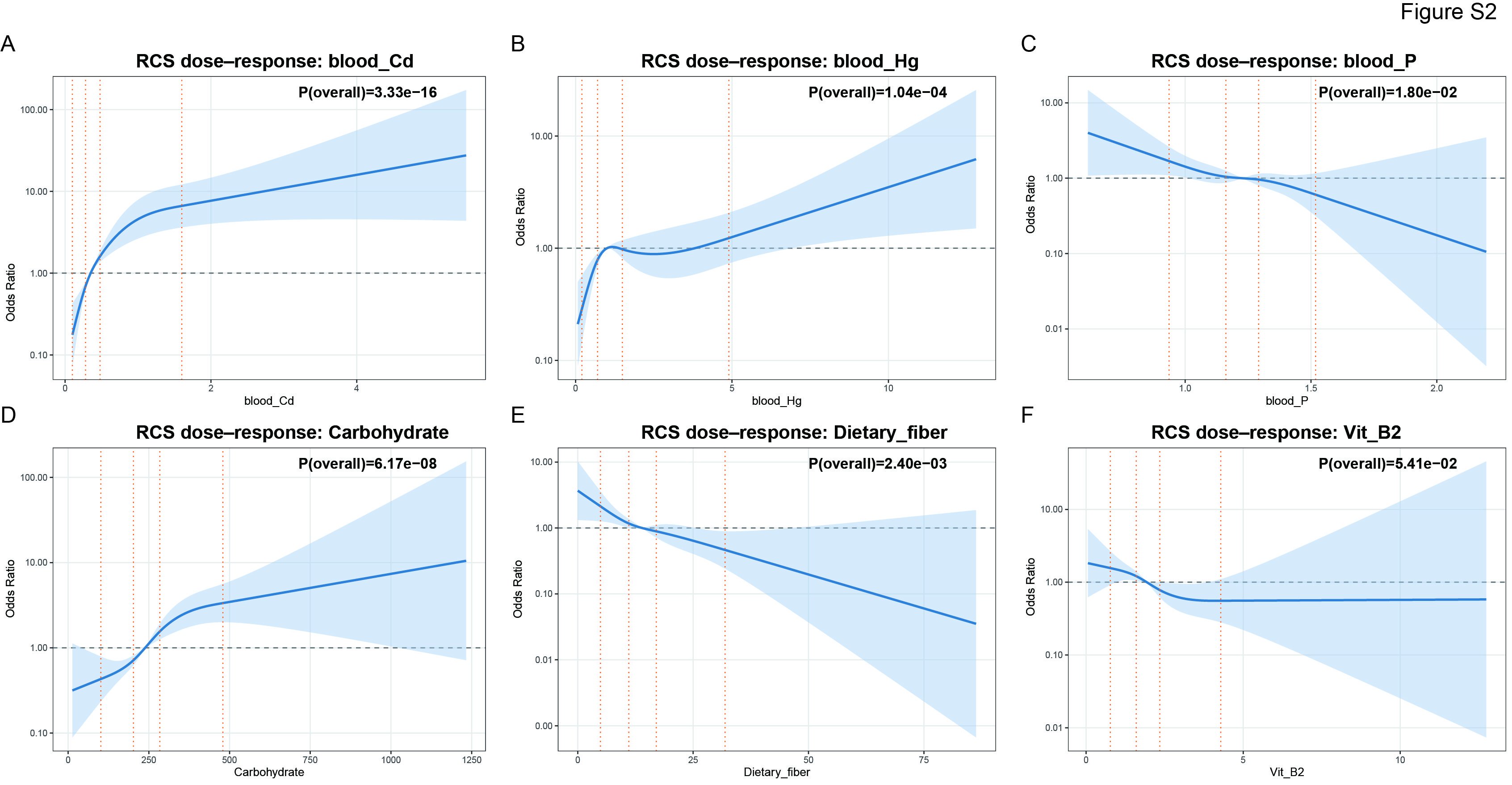

Supplement: Supplementary file 2 — Figure S2. Sensitivity analyses of restricted cubic spline models (K4). Sensitivity analyses of dose-response relationships using restricted cubic splines with four knots (5th, 35th, 65th, and 95th percentiles). (A) Blood cadmium (blood_Cd). (B) Blood mercury (blood_Hg). (C) Blood phosphorus (blood_P). (D) Carbohydrate intake. (E) Dietary fiber intake. (F) Vitamin B2 intake (Vit_B2) [file 12967_2026_8086_MOESM2_ESM.jpg]
